# Reply to Bertolaccini *et al.*: Small Nodules and Big Questions: A Closer Look at the RELIANT Trial

**DOI:** 10.1164/rccm.202506-1515LE

**Published:** 2025-08-04

**Authors:** Fabien Maldonado, Rafael Paez, Robert J. Lentz

**Affiliations:** ^1^ Division of Allergy, Pulmonary and Critical Care Medicine,; ^2^ Vanderbilt Interventional Pulmonary Research Laboratory, and; ^3^ Vanderbilt Center for Bioethics and Society, Vanderbilt University Medical Center, Nashville, Tennessee

*From the Authors*:

We thank Bertolaccini and colleagues for their thoughtful appraisal of the RELIANT trial and for their interest in our work. RELIANT was a single-center, open-label, pragmatic, cluster randomized controlled trial comparing the diagnostic yield of robotic bronchoscopy (RAB) to that of electromagnetic navigation bronchoscopy (ENB) (1). We appreciate the opportunity to clarify and contextualize several points raised.

First, a noninferiority design is used when there are advantages of a studied intervention, independent of efficacy, and all that is needed to support adoption of the preferred intervention is evidence that it is not significantly worse than the alternative. Despite limited comparative data on efficacy, RAB has been rapidly adopted in the United States on the basis of its ease of use, the stability of the catheter, and the potential for fine adjustment of the catheter tip during biopsies. The RELIANT trial provides reassuring evidence that these benefits do not come at the cost of diagnostic accuracy. Although Bertolaccini and colleagues raise concerns about the 10% noninferiority margin, the diagnostic yield of 77.8% in the RAB group, compared with 75.5% in the ENB group (a difference of 2.3% favoring the RAB group), would have been sufficient to demonstrate noninferiority at much more conservative thresholds.

Second, we agree with Bertolaccini and colleagues that subgroup analyses are not powered for interaction testing and that conclusions are, by definition, exploratory in nature. In line with CONSORT and ICEMAN recommendations, we reported confidence intervals without adjusting for multiplicity, which is reasonable when such analyses are intended to inform future research rather than guide definitive conclusions (2, 3). As suggested, we analyzed our data for nodules smaller than 20 mm: Without a bronchus sign (57 for ENB and 74 for RAB), diagnostic yield was similar between ENB (68.4%) and RAB (74.3%), with RAB meeting noninferiority criteria. Among nodules with a bronchus sign (40 in each group), ENB had higher diagnostic yield (75.0% vs. 67.5%), and RAB did not meet noninferiority criteria; however, the small sample size precludes strong inferences about clinical benefit.

Third, we acknowledge that the single-center nature of our study limits generalizability, and we reported this as a limitation in our article. We agree that future studies should prioritize external validity over internal validity to inform practice more broadly. We admit that performance may vary across institutions and operators; however, we believe that RELIANT provides a rigorous benchmark against which wider dissemination efforts can be measured.

Finally, RELIANT was intentionally designed as a pragmatic clinical trial, aiming to generate methodologically robust data in a timely fashion to inform patients care in a rapidly evolving technological landscape (4). The unprecedented pace of recruitment (411 patients in a year) was facilitated by embedding the study in usual care to maximize efficiency and minimize workflow disruption, and by the adoption of broad inclusion criteria, flexibility of cointerventions, and a primary outcome that is both patient centered and methodologically efficient (adjudicated shortly after the intervention) (5). Prescriptive demands altering usual care would have arguably interfered with recruitment and delayed data collection and analysis. Rather, we believe that RELIANT reflects real-word practice and represents an innovative paradigm for the evaluation of new technology, and we echo the call for integrated frameworks that consider not only diagnostic yield but also implementation feasibility, cost-effectiveness, and equitable access.
